# Promoting deceased organ donation: should familist incentives be adopted in Islamic regions?

**DOI:** 10.1007/s11017-026-09746-5

**Published:** 2026-03-03

**Authors:** Sanwar Siraj, Ruiping Fan

**Affiliations:** 1 Centre for Medical Ethics and Law, Medical Ethics and Humanities Unit, The University of Hong Kong, Hong Kong SAR, China; 2 Department of Public and International Affairs, City University of Hong Kong, Hong Kong SAR, China

**Keywords:** Familist incentive, Deceased donation, Organ transplantation, Islamic bioethics, Health law

## Abstract

This essay presents arguments for adopting a familist incentive approach for promoting deceased organ donation in Islamic regions. It argues that the familist incentive (a system in which the immediate relatives of a deceased donor are given priority in organ allocation within the waitlist of medically similar patients) should be adopted in Islamic regions, where the prevailing Islamic moral culture shapes the way of life for the majority of inhabitants by following the Islamic classics. The classics value the preservation of human life and legitimize the priority of saving the lives of family members. The essay also provides practical reasons to address objections to adopting such an approach in Islamic regions. It concludes that adopting a familist incentive approach could be both morally defensible and practically effective in these regions, thereby optimizing deceased organ donations, protecting the progeny of the family, and improving overall healthcare outcomes.

## Introduction

Organ transplantation is a modern medical intervention that saves the lives of patients with end-stage organ failure [1]. The transplantation process involves two main types of organ donors: deceased donors, who provide organs for transplants after death through established procedures, and living donors, who donate organs during their lifetime to help others in need. While some research indicates that living donor transplants have better medical outcomes for recipients compared to deceased donor transplants [2–4], it is essential to recognize that living donors face significant risks, including short- and long-term health implications stemming from surgical procedures, potential issues with the functioning of the remaining organ, and psychological distress in post-donation periods [5]. Thus, it is generally agreed that promoting deceased donation is essential, while acknowledging the importance of living donation. This essay will focus exclusively on legitimate incentives for deceased donation.

While deceased organ transplants offer hope and improved quality of life for recipients, the demand for posthumous organs, such as kidneys, far exceeds the available supply. For example, the actual deceased donation rates per million population (pmp) in the three countries with the highest donation rates in the world in 2023 were as follows: 49.38 (Spain), 48.04 (US), and 36.80 (Portugal) [6]. Nevertheless, organs for transplant remain insufficient for these countries. For example, an average of 17 patients on the waiting list die each day without having transplants in the US [7]. Situations in other countries and regions are worse than these three. In most Islamic countries, deceased donation rates are extremely low. For example, the actual deceased donation rates pmp in 2023 were as follows: Iran at 12.57, Kuwait at 7.67, Qatar at 7.41, Saudi Arabia at 4.00, Turkey at 3.58, and Bangladesh at 0.01 [6].

Various types of incentive measures have been adopted worldwide to promote deceased organ donations [8–11]. There are three major types of incentive measures, namely honorary, compensationalist, and familist. An honorary incentive is a form of non-material reward or appreciation provided to the donor’s family, such as a medal of honor, a thank-you letter, a memorial park, and other similar gestures. A compensationalist incentive is a form of financial support in which either the government or the recipient provides monetary compensation to the family of a deceased donor for organ donation. A familist incentive is a registry system where the close relatives of a deceased donor are given priority in organ allocation within the waitlist for organ transplantation.^1^ Comparatively, an honorary incentive is morally uncontroversial but practically insufficient^2^; a monetary incentive is practically effective but morally controversial,^3^ and a familist incentive is effective but remains underdiscussed [5]. This essay will only focus on familist incentives.^4^

We attempt to defend adopting familist incentives in Islamic regions. By “regions,” we mean jurisdictional areas within countries or states where the prevailing moral culture influences the lifestyles of the majority of inhabitants, even if some individuals do not align with that culture. We recognize that in a large country, different dominant cultures are present in different regions. When we use “Islamic regions,” we refer to areas where people adhere to Islamic religious and moral beliefs, and where Islam is practiced as the dominant moral culture, without assuming that all regions in a so-called “Muslim country” possess Islamic culture as their prevailing culture. We argue that Islamic regions should adopt familist incentives to enhance their deceased organ donation.^5^

In the next section, we present the familist incentive approach for deceased organ donation prevalent in cultural regions from the Middle East to the Far East, including at least one Islamic country, the Islamic Republic of Iran. Iran has, in fact, already adopted the familist incentives enhancing deceased donation, although this is not well-known as its monetary kidney donation incentive model for living donation. The following section highlights Islamic classical familist ethical foundations and moral reasoning to defend adopting this type of incentive for deceased donation in Islamic regions. Then we address several potential objections to adopting familist incentives, including concerns over altruism, individual autonomy, potential coercion inside the family, and favoring large families, providing responses to them. The final section offers concluding remarks, summarizing that adopting a familist incentive approach could be effective and morally permissible in Islamic regions, enhancing deceased organ donations and improving overall healthcare outcomes.

## Familist incentives: from the Middle East to the Far East

Israel in the Middle East was the first country to implement the familist incentive measures to boost organ donation rates. Initially, Israel faced a challenge with low organ donation rates due to Jewish law prohibiting the violation of the dead. In response, the Israeli government introduced a groundbreaking approach by incorporating “nonmedical” criteria into a “priority system” based on medical criteria. The Organ Transplantation Law was passed in 2008, publicized in 2010, and fully enforced in 2012 [30–32]. Under this law, registered donors receive priority for organ allocation if they ever need one, with three levels of priority defined for living and deceased donors’ families. The first priority is granted to candidates whose first-degree relatives donated organs after death or who have registered as organ donors themselves. The second priority is for candidates who have been registered as donors for at least three years, and the third priority is for candidates with registered first-degree relatives. The law was subsequently amended to include any living donor who previously donated organs such as a kidney, liver lobe, or lung lobe, granting them first priority in organ allocation [33].

This approach was rooted in the cultural value of showing love and special treatment to family members in Israel, driving the prioritization of transplant waiting lists for those connected to deceased organ donors or previous organ donors. An extensive public awareness campaign was launched to educate the population about organ donation, utilizing various media platforms and outreach strategies. The new law also provided fair compensation for living donors, covering lost wages and related expenses [30]. This resulted in an overwhelming response from the Israeli population. Seventy thousand Israelis registered for organ donation cards within the first 10 weeks of the campaign [34]. The impact of the new organ donation policy in Israel was evident in the data. In 2011, the organ donation rate in Israel saw a significant increase from 7.8 to 11.4 donors pmp, reflecting the success of the incentive measures implemented [33]. The outcome of donations has been significantly improved, and the consent rate by families for deceased organ donation has been increasing [35, 36]. This positive outcome underscored the effectiveness of familist incentives in motivating individuals to become organ donors and facilitating a culture of organ donation within the Israeli population [37].

Iran, another country in the Middle East, has also adopted familist incentives for organ donation. There is no evidence to show that Iran has been inspired by Israel in this regard. In fact, Iran’s financial support for living kidney donation is worldly well-known and highly controversial [14, 38], but its policy of adopting familist incentives for deceased organ donation has been broadly overlooked. Familist incentive approach is extended to both deceased and living organ donations in Iran. Specifically, incentives are offered to live donors in the event of kidney failure in their future life and to first-degree relatives of brain-dead deceased donors, as initiated by the Iranian Ministry of Health [39]. The introduction of these incentives for brain-dead donors’ first-degree family members coincided with the launch of Iran’s Brain-Dead Donation (BDD) program by the Ministry of Health. In Iran’s biomedical practice, as in many Muslim societies, family consent post-death is deemed essential (a soft opt-in system), even if the deceased had provided consent before passing or had a registered donor card. Therefore, while the deceased’s consent is important, the family’s approval at the time of brain death is a crucial requirement for organ donation to proceed, irrespective of prior consent or donor card registration. The prioritization of receiving organs in need can serve as a motivating factor for families to consent to organ donation, with cultural, educational, ethical, social, religious, and belief-related factors playing significant roles, particularly in the context of brain-dead donations. First-degree relatives, such as parents, spouses, and children, are considered deserving of community recognition if they require an organ, as their consent to donation in a challenging situation reflects humanitarian and altruistic intentions, demonstrating empathy towards others [39].

The adoption of familist priority measures in Iran has not only provided emotional support to Iranian families by acknowledging their legitimate concerns but has also contributed to the public awareness of the brain death criteria. The Iranian government covers the direct transplantation expenses for brain-dead donor screening, transfer, management, organ retrieval in organ procurement units, and transplant operations in transplantation centers [40]. Consequently, public understanding of brain death has improved over time, leading to a rise in kidney transplants from brain-dead donations from zero in 2000 (when the BDD act was introduced) to 18 pmp in 2019, with a corresponding decrease in live kidney donations [41]. In 2019, kidney transplants were performed in 32 transplant centers in Iran, with 1354 from deceased donors and 747 from living donors [42].

Israel’s familist incentive model has inspired some regions in the Far East to follow, such as mainland China and Taiwan. These regions carry long-standing Confucian family-oriented traditions in their societies. In addition to the honorary and compensationalist incentive measures [43], China has also adopted familist incentives in the posthumous organ donation program, where family members of deceased donors who have successfully donated organs receive priority if they require an organ for transplantation.^6^ This priority incentive is outlined in the official document “The Basic Principles of Human Organ Allocation and Sharing in China and Core Policy on Liver and Kidney Transplantation” issued by the Chinese Ministry of Health in 2010 [45]. According to this policy, living donors or immediate family members of posthumous donors have a reasonable priority right in the allocation of donated vital organs, such as livers or kidneys, when needed. In 2018, the National Health Commission issued a notification to revise the basic principles and core policy for liver and kidney transplants and formulated a core policy for the allocation and sharing of hearts and lungs [46]. While many provinces in China have followed this directive and incorporated it into their organ donation regulations, the specifics of the family priority incentive may vary among regions. The scope of family members eligible for priority varies among provinces, illustrating inconsistencies in policy implementation. For example, Shandong’s regulations grant preferences to close relatives for the clinical use of human tissue, while Heilongjiang Province and Chongqing City limit the scope of priority to the donor’s spouse, parents, and children, excluding siblings, grandparents, and grandchildren. While some provinces have included family priority incentives in their regulations, a consistent national-level confirmation of this incentive is yet to be established [47].

The introduction of familist incentive measures may have served as a significant factor contributing to the growth of voluntary organ donation in Mainland China. Since 2015, China has experienced a significant increase in voluntary organ donation. By 2018, the number of deceased organ donations and the rate of donation pmp had risen substantially, reflecting the positive impact of the incentive measures adopted, including the familist incentive measures. The number of deceased donations pmp in China increased from 2,766 (2.01%) in 2015 to 6,303 (4.53%) in 2018 [48].

Following the example set by Israel [49], Taiwan has implemented organ allocation legislation that prioritizes candidates if a family member has previously donated an organ [50]. The legal framework governing the transplantation and allocation of human organs in Taiwan, established in 2014, specifies that waitlisted candidates will be given priority if a close family member (ranging from first to third degree) has been a past organ donor [50]. Taiwan’s new policy of organ distribution assigns family priority, allowing individuals to receive credits to speed up the process of obtaining a transplant organ if a family member has donated organs in the past. Taiwan has steadily increased organ donation rates through effective legal frameworks and public education programs. As a result of these efforts, deceased donors have increased steadily from 6.7 pmp in 2005 to 15.2 in 2019,^7^ including single tissue donation [53, 54]. This improvement, we believe, is attributed, at least in part, to the implementation of familist incentive policies.

## Islamic ethical foundations for familist incentives

Islam is a comprehensive way of life that provides guidance for daily living for Muslim believers, including healthcare decisions that shape public policies [55]. Muslim believers adhere to the principles of classical Islamic teachings, such as the Quran and Sunnah. Islamic teachings prioritize saving human lives over death. The necessity to save human lives makes organ donation permissible [56–60]. Islamic teachings encourage Muslims to be compassionate towards all humankind (Quran 5:32), while also emphasizing the importance of actively assisting family members. Muslims should protect family members through donations before offering such support to others. It should be recognized that family relationships are a fundamental aspect of ethical behavior in Islamic regions. Muslims have a mutual belonging that is a defining characteristic of a family, and the sense of belonging forms the basis of relationships within families in Islamic society [59].^8^

The Quran specifically highlights the significance of family ties (*Silat al-Rahim*), stating that Muslims who are connected by blood relations are closest to each other (8:75). This emphasis serves as an ethical foundation for family relations within Islamic law. A family member deserves more rights and care in Islamic regions. The Almighty instructs Muslims to maintain kinship ties (Quran 4:1). For example, if a family member and a stranger are both in critical conditions that need aid, family members deserve more right to get it from their relatives [62]. A family priority right is specified in the Quran. The Quran specifically mentions, “And give to the near of kin his due and [to] the needy and the wayfarer, and do not squander wastefully” (17:26). This verse implies that Muslim believers should extend their care and assistance to their family members first, even through organ donations. The prioritizing of assistance to family members is also reinforced in another verse of the Quran (16:90). Offering organs to family members in times of need is a kind of one’s moral duty to support each other.

The teachings of Prophet Muhammad highlight the moral obligation to prioritize family members in times of need, and this should reasonably be interpreted as including when faced with critical conditions such as organ failure. A teaching attributed to Prophet Muhammad underscores the importance of family ties, stating that “the best beloved of God is one who loves his family and kin the most” [63, pp. 30–31, see also 64, p. 30]. This Prophetic tradition emphasizes the significance of showing compassion and prioritizing the well-being of one’s own family members. If someone saves a life by donating organs, it is a kind of love offering that ensures well-being, as the person loves their Almighty most. This is a moral commitment to supporting family members that reflects the core values of Islam, which emphasize love, compassion, and care for one’s kin. Giving proper due to a family member is also emphasized by the Prophet, saying, “Do not ever sever your relationship with a member of your family even if he severs his relationship with you” (Bukhari and Muslim).

Islamic teachings prioritize familist values and give preferential treatment to one’s family members. Our relevant question is, who should get preference if a close relative and a distant relative need organs for transplantation? The Quran has specific verses about who should get priority in receiving support and assistance. The Quran mentions that Muslims should offer something good to parents first, then to relatives, and next to orphans, the needy, the neighbors near and far, to travelers in need, and to slaves (4:36). Another verse mentions a similar emphasis, “Whatever you should give are for parents, relatives, orphans, the poor, and needy and travelers” (Quran 2:215). If we apply the meaning of these Quranic verses to organ donation issues, it specifies that if a person needs organs for life-saving treatment, it is his duty to protect his parents, relatives, and then others.

Islamic teachings allocate graded love for each family member who deserves preferential support and care. The support and assistance provided within family relationships are primarily guided by the specific family relations between individuals. Islamic law emphasizes caring for close relatives, which carries a greater reward than assisting more distant relatives. Family members receive more support and care based on the order described in Islamic teachings. The Quranic verses (2:177; 17:23; 24:22) state that parents are given the foremost place in receiving care and assistance, followed by other close relatives such as sons, daughters, brothers, sisters, and other adult siblings who are blood-related, as well as other non-immediate relatives. This order of relationships holds significant importance in Islamic law, where each individual receives precedence over later persons. Islamic law emphasizes providing greater care and support to close relatives, such as mother, father, son, daughter, brother, and sister, compared to more distant relatives, such as uncles and aunts from both paternal and maternal sides. Recognizing Islamic ethical principles that prioritize care for close relatives over distant relatives, and remote relatives over unrelated individuals, could extend to deceased organ donation policies and medical practices. These ethical foundations are more relevant to or supportive of adopting familist incentives for promoting deceased donation in Islamic regions.^9^ It is reasonable to argue that patients with a first-degree relative who was a deceased donor should be given priority to receive organs for transplantation.^10^ The close genetic relations and kinship ties may encourage family members to become deceased donors by donating organs to immediate family members for transplantation.

Accordingly, we argue that all Islamic regions should adopt familist incentives for deceased organ donations. There are at least two reasons to support this proposal. First, this proposal could be supported by the Islamic familist ethical foundations as outlined above. Second, it could also contribute to promoting deceased organ donations, as one useful measure, in these regions.^11^

For example, the Bangladesh government passed the Human Organ Transplantation Act in 1999, which was subsequently revised in 2018 to offer priority access to deceased organs for family members in need of transplantation. Under the revised Act, certain recipients are given priority for receiving organs from deceased donors for transplantation. These include individuals who have given written consent for organ donation to a close relative or another person during their lifetime, younger individuals, those near death where the procedure could potentially save their lives, and individuals who are geographically nearby or have a short travel time to the transplantation facility (Sect. 7c:3) [81]. This provision in the revised law aims to provide equal opportunities for all individuals in need of organ donation while prioritizing the wishes of the brain-dead donor’s family. By giving precedence to family members for receiving organs from deceased relatives, the government of Bangladesh aims to promote deceased organ donations. Adopting a familist priority approach might promote deceased donation rates in Islamic regions while providing familial consent for organ retrieval from deceased relative donors. While living donations have been common in Islamic countries, including Bangladesh, the transplantation of vital organs from posthumously donated organs has been limited.

A significant milestone was achieved in Bangladesh on January 19, 2023, when a young female battling an incurable disease was declared brain dead [82]. Following her wish to donate her organs to needy recipients, including remote relatives (as there were no immediate family members requiring transplants), her mother consented to the retrieval of her two kidneys and two corneas for transplantation, which were then transplanted to four unrelated individuals in need. This case illustrates the importance of respecting an individual’s wish to prioritize relatives for using their organs in the context of deceased organ donations for transplantation. Adopting a familist priority approach in Islamic regions could promote organ donation rates and ensure the well-being of those family members in need of life-saving transplants.

## Responses to potential objections

### Altruism-based concerns

Scholars commonly argue that organ donation should be viewed as an unselfish and altruistic act based on an egalitarian approach. Some critics state that giving preferences to one’s family members could be perceived as selfish, and offering family priority in organ allocation may not be regarded as morally acceptable [83, 84]. If adopted, the critics may contend, this would change the moral nature of the act of donation and make it no longer altruistic.

In response, we agree that organ donation should be an altruistic act, characterized by behavior that benefits another individual at a personal risk or cost. Altruism typically involves selfless concern for the well-being of others, with individuals intentionally aiding others for their benefit. However, although “altruism” itself suggests that donations should aim to benefit others rather than oneself or family members, we should recognize the legitimacy of “mixed altruism” in human behavior [9]. Mixed altruism acknowledges that individuals may be motivated by a combination of selfless and self-interested reasons when making decisions, such as in the case of organ donation under familist incentives. In this context, the act of donating organs to benefit family members can be seen as a form of mixed altruism, where both altruistic and self-interested motives coexist. Prioritizing family members should not be taken as excluding the fact that there is a genuine disinterested motive to benefit others through donation, alongside a desire to help family members. In addition, the consequences of increasing deceased donations under familist incentives are beneficial to others, as one’s family members may eventually not need organs. Accordingly, although deceased donations under familist incentives may become mixed-altruist rather than purely altruistic, they remain altruistic and should be defended.

In the context of Islamic regions, where family values and kinship are highly esteemed, prioritizing family members in organ donation can be seen as a way to promote the well-being of the community as a whole while respecting Islamic teachings, ensuring “greater good” for family members. Anybody can direct their organs towards whom they feel a strong sense of moral obligation and solidarity [85]. This kind of altruism may evoke one’s strong feelings or close attachments that one may not share with everyone. One could be willing to donate organs, seeing that their organs might benefit other family members. Adopting familist incentives can be viewed as an effective strategy to enhance donation rates and save more lives in Islamic regions. The presence of both altruistic and self-interested motives does not diminish the ethical value of the donation, as long as the primary intention (*niyyah*) is to benefit others, including one’s family members. Implementing a family priority approach through policy reforms could potentially stimulate increasing organ donation rates ethically in Islamic regions, which ultimately benefits the whole society.

### Individual autonomy-based concerns

Some scholars have expressed concerns regarding individual autonomy within the context of familist incentives in deceased organ donations. The central issue raised by them is how to uphold the principle of respect for autonomy in organ donation as an individual choice while adopting familist incentive measures. They worry that with such measures implemented, individuals may not be able to autonomously make their donation decisions [86].

In fact, a more communitarian than individualistic cultural features generally characterize most muslim families in Islamic regions. It is the family where all individuals reside together and make everyday decisions together, including those related to healthcare [87]. All members openly share their opinions and engage in discussions, and contribute to final decisions within the family structure. These discussions consider both individual preferences and family concerns, fostering a supportive environment for reaching collective decisions. Families consider all individual wishes and preferences and make decisions that are good for the welfare of the family as a whole. A quasi-traditional view of the family is still vibrant in contemporary muslim families. In this view, the family is seen as a close-knit moral community, taking care of every member’s welfare, and every member should be involved in decisions about important issues (such as organ donation) collectively with other members. This means such decisions are normally shared familial decisions rather than individual and independent decisions.

The communitarian features prevalent in muslim families are a defining characteristic of decision-making processes concerning deceased organ donation in Islamic regions. This can be described as a familist decision-making model, where families in Islamic communities possess legitimate authority to decide on organ donation for deceased family members who did not register for donation during their lifetime. Conversely, families also have the legal power to veto a deceased family member’s organ donation, even if the individual had formally registered with the donation registry system. It is explicit that the family’s consent is essential in Islamic regions before organ retrieval for transplantation, even if the deceased had expressed a desire to donate organs posthumously. These dual powers held by families—to donate the organs of non-registered family members and to override a deceased family member’s wish to donate—form the two pillars of the familist decision-making model for deceased organ donation in Islamic regions. This approach underscores the significance of family dynamics and collective decision-making traditions within Islamic societies, where family is prioritized in healthcare decisions, including organ donation, while individual choice counts less.

### Concerns about potential coercion within the family

Some have raised concerns that familist incentives may create coercion within families. They caution that implementing familist incentives could lead to situations where a patient’s immediate family members might exert undue pressure on the patient to sign up for deceased donation to secure priority for themselves. There is also a risk that family members may choose to donate the organs of a deceased loved one against their explicit wishes not to donate. Therefore, under familist incentives, families may make decisions that serve their own interests, even if they contradict the known wishes of the patient regarding organ donation. Individuals, especially those who are less advantaged within a family, may even be coerced into donating to “benefit” other members [84].

In response, we should first note that in Islamic culture, it is not ethically impermissible for a family to encourage one of their members to register as a deceased donor, as long as the discussion is free from coercion or manipulation. For example, if a family engages in a conversation with a terminally ill member and encourages them to agree to donate after death through persuasion, there is nothing ethically problematic about this. In fact, this should be seen as ethically admirable because organ donation can save lives. Even if part of the motivation for family members is to benefit from the priority right generated by the donation under familist incentives, the involvement of these incentives does not change the moral nature of the decision when based on mutual consent and persuasion rather than coercion.

The possibility that some families may choose to donate a deceased family member’s organs for their own benefit under familist incentives cannot be completely eliminated. Many Islamic countries have adopted a soft opt-in system where one may register their wish to donate organs after death. Even for unknown wishes of organ donors, in practice, the family has the legitimate right to make a surrogate decision for donation, even if the deceased member has not registered as a donor. However, the more common concern in Islamic regions is families vetoing donations, even when the deceased had expressed a desire to donate, due to reasons such as the religious stance to keep the deceased body intact (Quran 75:3–4), as organ donation requires breaking Islamic religious restrictions against cutting the human body to preserve a life (Quran 2:173; 5:32). This situation often arises when the donor’s wishes remain unknown to the family. Families may not want to proceed with such donations because they fear potential consequences in the afterlife for what they perceive as wrongdoing.

One challenge in Islamic regions is the lack of discussion between donors and their families regarding organ donation. It is unlikely that families would decide to donate the organs of deceased family members against their wishes. The human body is a short trusteeship from the Almighty, and human life and the body should be protected and cared for. Coercion is seen as a wrong deed, and the Quran instructs Muslims to help one another in what is good and right. The Quran (5:2) emphasizes the importance of cooperation in “righteousness” and “piety” while avoiding “sin” and “transgression”, encouraging Muslims to be potential organ donors who can be collaborative and serve righteous purposes in their families. Still, to prevent possible abuses resulting from familist incentives, it would be crucial for the public to understand that everyone has the right to decide not to donate. Individuals should communicate their decision clearly to each immediate family member to ensure their wishes are respected. This explicit announcement would remind those who do not wish to be donors of the importance of expressing their wishes. Emphasizing the care for every family member’s interests, including preserving a deceased member’s body, is important in Islamic teachings and can prevent a family from making a surrogate donation decision against a deceased member’s wishes.

### Concerns about favouring large families

Some may raise concerns that familist incentives for organ donation may favor large families and create unfair advantages for individuals with more first-degree relatives [88]. Critics may argue that rewarding family members for another person’s contribution and excluding those without immediate family members from priority access to organs is unjust and inappropriate.

In response, we think that these policies can be ethically justified by drawing on Islamic ethical principles and the traditional values that emphasize the importance of family ties and mutual care. The prevailing quasi-traditional view of the family in Islamic regions underscores the significance of familial relationships as a central aspect of moral values, aligning with Islamic teachings that promote compassion and support among family members.

In Islamic societies, family relationships carry deep moral values and obligations. This is why they provide preferential life-saving treatment to family members. Islamic teachings clearly specify that a muslim is not entitled to inherit from a non-Muslim, and vice versa. Only muslims have legal rights to receive inheritance from their legal heirs. They exemplify the distribution of inheritance to prioritize family members. If a person passes away, leaving an inheritance, it is distributed among their rightful heirs, such as sons, daughters, and wives (Quran 4:11). Only close family members deserve legal rights to receive an inheritance. Inheritance laws reinforce the values of familial relationships and strong kin ties. In this context, offering familist incentives for organ donation can be viewed as a fair practice within the inheritance framework. Just as it is deemed fair for family members to have priority in receiving financial support or inheritance, it is also considered fair for them to have priority access to organs if needed, because of organ donation within the family. While some may argue that familist incentives may disadvantage individuals without immediate family members or those from smaller families, proponents assert that the overall goal of increasing organ donations to save lives justifies the policy. The aim is not to unfairly advantage certain groups but rather to enhance the availability of organs for transplantation, benefiting individuals in need regardless of their family size or structure. Consequently, even those without family members will benefit from the familist incentives, because more deceased donations will be accomplished under this policy.

## Conclusion

In this essay, we propose the adoption of a familist incentive approach to deceased organ donation in Islamic regions. This approach could grant priority to patients for organ needs with an immediate family member who had been a deceased donor. This proposal is not for the global community but is limited to Islamic regions, guiding organ transplantation practices. This is not because we do not believe this incentive should be universally adopted. Rather, our arguments have been based on the reasons offered by Islamic tradition and considering the social and medical conditions of Islamic regions. It is reasonable and legitimate for contemporary Islamic societies to adopt this familist incentive approach to improve deceased organ donations. This proposal assumes that culturally appropriate medical regulations should be determined by particular regions based on their respective mainstream moral cultures. The familist incentive approach is both ethically defensible and predictively useful in Islamic regions.

## Data Availability

Data sharing not applicable as no datasets were generated and/or analyzed for this study.

## Footnotes

1 Under this system, when a person donates their organs after death, their immediate family members—such as parents, siblings, or children receive preferential placement on the transplant waiting list if they require an organ. This is not referring to a situation where one family member needs an organ transplant while another eligible family member who has died is a good match for donation. Instead, it is a priority tiering structure that assigns different levels of priority to patients based on the closeness of their relationship to previous deceased donors, as illustrated by the system in Israel, discussed in the next section. If such a system can enhance much more deceased donations, it would significantly benefit those patients having relatives being deceased donors because with priority given in the waiting list, they can more easily find a good medical match of donated organs.

2 When we say that an honorary incentive is “practically insufficient,” we do not mean it is ineffective. However, it may not provide enough motivation for a significant increase in donation rates in most societies. While many individuals may appreciate honorary recognition, this alone may not be sufficient to overcome fears, cultural reservations, or a lack of awareness surrounding organ donation [5, 12, 13].

3 Iran provides a well-known example of monetary incentives for living kidney donation, which has been both effective and controversial [14, 15]. Many argue that paying for organs risks exploiting vulnerable populations, commodifying the human body, and undermining the altruistic foundation of donation systems. Additionally, there are concerns that monetary incentives could create inequities in access to transplants, favoring those who can afford to pay or those who feel economically pressured to donate [16–19]. These moral controversies have prompted many countries and international organizations to prohibit or strictly regulate monetary incentives for organ donation [20–23].

4 Familist incentives leverage existing family bonds and social obligations, which can be particularly powerful motivators in cultures where family ties are strongly valued, such as many muslim communities. Ethically, familist incentives avoid the commodification issues associated with monetary incentives, as they do not involve financial transactions but rather a prioritized allocation within the existing donation framework. Despite these advantages, familist incentives have not been widely explored or implemented, possibly due to concerns about fairness in organ allocation or lack of awareness about their potential benefits.

5 While most regions in Bangladesh, for example, are predominantly Islamic, the Chittagong Hill Tracts—comprising Bandarban, Rangamati, and Khagrachhari—are majority Buddhist. We believe that each region should have the moral authority and right to formulate their ethical policies on organ donation in accordance with their mainstream culture. In principle, a religious minority region should not be compelled to participate in a national transplantation system for the general medical benefit of matching organs across regions, even if their refusal might lead to medical disadvantages, such as only having access to their own organs. Fortunately, Buddhists in the Chittagong Hill Tracts may not hold such a refusal. Although Buddhists and muslims have differing metaphysical views regarding the body and the integrity of the body post-death [24–26], both groups emphasize the preservation of human life and the reduction of suffering. This shared commitment can foster support for voluntary organ donation as a means to achieve these values [27–29]. Any disagreements about the meaning of the body can be addressed in different ways between the regions involved during the organ donation process, respecting diverse perspectives. In short, this Buddhist region should not be required to adhere to Islamic ethics but should be encouraged to make bioethical decisions based on their own values while engaging in communication and negotiation with other regions and the national government.

6 The implementation of the family priority incentive varies across provinces and major cities in China, with different policies and regulations governing the prioritization of family members for organ transplantation. The diverse approaches to family priority incentives highlight the complexities and variations in organ donation policies across different regions in China. Some provinces and regions in China like, for example, Zhejiang, Tianjin, Jiangxi, Jinan, and Liaoning have introduced financial compensation policies, resulting in an increased number of organ donors [44].

7 Organ transplant rate in Taiwan is currently stagnant, standing at 5.85 pmp in 2023 [51]. One cause for this is the challenge in determining brain death. Many patients with catastrophic injuries do not meet the neurological criteria for brain death or experience cardiac arrest before organ donation, leading to only tissue donation being possible from the donor [52].

8 Islam is not a monolithic faith; it encompasses various sects [61]. The two main sects are Sunni and Shia. Geographically, the majority of Shia communities reside in Iraq, Bahrain, and Azerbaijan, while Sunni Muslims are predominant in many other countries, such as Bangladesh, Egypt, Indonesia, Jordan, Malaysia, Pakistan, Turkey, the UAE, and Saudi Arabia. Despite disagreements on various issues, both traditions emphasize the importance of preserving human lives and honoring family obligations [59]. Both sects can agree on Islamic ethical foundations and familist incentives for deceased organ donation, as saving the lives of family members is highly prioritized in Islamic texts, such as the Quran and Sunnah. Accordingly, in this paper, we focus on these common ethical foundations and family-centered rationales for deceased organ donation across all muslim sects, without delving into sect-specific doctrinal differences.

9 Some may argue that in many Islamic regions, members of a husband’s family may be reluctant to donate to members of the wife’s family, and vice versa. However, regarding deceased donations, studies have shown that in many countries, including muslim countries, men are more frequently the donors than women [65, 66]. Although some research suggests that women may be more willing to donate in certain contexts [67, 68], societal factors and cultural norms often result in men being the majority of deceased organ donors [69, 70]. In contrast, when it comes to living organ donation, the situation is often reversed, with women more frequently donating organs, such as kidneys or liver lobes to their relatives [71–75]. Various reasons contribute to this pattern, including a higher prevalence of certain diseases among men, women’s roles in household responsibilities, potential immune sensitization related to childbearing, and social obligations, altruism, and empathy displayed by female donors [76–80]. In our view, as long as women voluntarily donate their organs for their family members, such practices are morally acceptable.

10 Adopting such family incentives is not only morally fair according to Islamic values, but it is also beneficial for everyone involved. The immediate family members of a deceased donor may not ultimately need any organs. Therefore, even if the donor was primarily motivated by familial intentions, their donations would increase the organ pool and benefit others in need.

11 We do not have epidemiological data from Muslim-majority countries to directly support the claim that familial incentives will increase organ donation rates in Islamic regions. This paper focuses on a conceptual discussion, drawing on evidence from non-Islamic regions, such as Israel, mainland China, and Taiwan which demonstrate that the adoption of family-based incentive measures has led to increased organ donation rates. Based on this evidence, we can reasonably predict that implementing a familist incentive approach could similarly be effective in boosting organ donation rates within Islamic regions, given that the Islamic ethical foundations emphasize a moral responsibility to care for family members.
